# Detection and Discrimination of Non-Melanoma Skin Cancer by Multimodal Imaging

**DOI:** 10.3390/healthcare1010064

**Published:** 2013-10-17

**Authors:** Sandro Heuke, Nadine Vogler, Tobias Meyer, Denis Akimov, Franziska Kluschke, Hans-Joachim Röwert-Huber, Jürgen Lademann, Benjamin Dietzek, Jürgen Popp

**Affiliations:** 1 Institute of Photonic Technology Jena (IPHT), Albert-Einstein-Straße 9, Jena 07745, Germany; E-Mails: sandro.heuke@ipht-jena.de (S.H.); nadine.vogler@uni-jena.de (N.V.); tobias.meyer@ipht-jena.de (T.M.); denis.akimov@ipht-jena.de (D.A.); benjamin.dietzek@uni-jena.de (B.D.); 2 Department of Dermatology, Venerology and Allergology, Charité-Universitätsmedizin Berlin, Charitéplatz 1, Berlin 10117, Germany; E-Mails: Franziska.Kluschke@gmail.com (F.K.); joachim.roewert@charite.de (H.-J.R.-H.); juergen.lademann@charite.de (J.L.); 3 Institute of Physical Chemistry and Abbe Center of Photonics, Friedrich-Schiller-University Jena, Helmholtzweg 4, Jena 07743, Germany

**Keywords:** CARS, TPEF, SHG, multimodal imaging, coherent Raman scattering, skin cancer, basal cell carcinoma, squamous cell carcinoma, non-melanoma skin cancer

## Abstract

Non-melanoma skin cancer (NMSC) belongs to the most frequent human neoplasms. Its exposed location facilitates a fast ambulant treatment. However, in the clinical practice far more lesions are removed than necessary, due to the lack of an efficient pre-operational examination procedure: Standard imaging methods often do not provide a sufficient spatial resolution. The demand for an efficient *in vivo* imaging technique might be met in the near future by non-linear microscopy. As a first step towards this goal, the appearance of NMSC in various microspectroscopic modalities has to be defined and approaches have to be derived to distinguish healthy skin from NMSC using non-linear optical microscopy. Therefore, in this contribution the appearance of *ex vivo* NMSC in a combination of coherent anti-Stokes Raman scattering (CARS), second harmonic generation (SHG) and two photon excited fluorescence (TPEF) imaging—referred as multimodal imaging—is described. Analogous to H&E staining, an overview of the distinct appearances and features of basal cell and squamous cell carcinoma in the complementary modalities is derived, and is expected to boost *in vivo* studies of this promising technological approach.

## 1. Introduction

At present non-melanoma skin cancer (NMSC) accounts for about 20% of all newly detected malignant neoplasms in humans [1]. At the beginning of the 21st century, the number of annual incidences of NMSC varied between 50 and 2,000 among 100,000 individuals in Finland and Australia [2]. Still, the number of incidences rises—e.g., by 4.2% in the USA every year between 1992 and 2006 [3]. The explanation for the rise of skin cancer incidences can be found primarily in the altered recreational behavior and the concomitant UV-liability. However, also the increased life expectancy together with the more complete registration of skin cancer incidences contributes to this trend [1].

Fortunately, the mortality rate for non-melanoma skin cancer remains low compared to other types of cancer [4]. This can be ascribed to a low metastatic potential, but also the efficient and effective ambulant treatment, which is possible due to the exposed position of skin cancer. The precondition for the appropriate treatment, however, remains the detection of the skin neoplasm and its accurate pre-surgery classification. Additionally, it has to be considered that even though the post-surgery classification is correct, numerous excisions are performed unnecessarily. The sensitivity for clinical diagnosis of NMSC is estimated to be about 56% to 90% while the specificity is in the range of 75% to 90% [5]. 

In order to improve the pre-surgical accuracy of classification and to avoid unnecessary invasive treatments, a number of tools for the cancer diagnostic were developed, e.g., optical coherence tomography (OCT) [6,7,8], magnetic resonance imaging (MRI) [9] and high frequency ultrasound (HFUS) [10]. These—clinically established—imaging techniques often do not provide the necessary spatial resolution for the diagnosis of early stages of skin cancer [11]. Hence, the “gold standard” of skin cancer diagnostic—the microscopic bright field observation of stained tissue—has remained unaltered for decades. This examination is preceded by the—intrinsically invasive—tissue excision and subsequent sectioning. The procedure is not only stressful for the patients, but also causes an unavoidable time delay between examination and diagnosis. 

Over the last two decades, *in vivo* microscopy was developed as a new approach—changing the sequence of steps from the established procedure, *i.e.*, performing *in vivo* microscopy before tissue excision. In this context *in vivo* two-photon excited fluorescence (TPEF) and second harmonic generation (SHG) are combined to map the distribution of endogenous autofluorophores and collagen in skin. The combination of SHG and TPEF has been successfully applied *in vivo* and *ex vivo* to a variety of dermatology-related questions [12,13,14] ranging from transcutaneous drug penetration [15] and monitoring dermal structural features [16,17,18] to tissue alterations, such as photoaging and skin diseases [19,20]. 

Among the investigated diseases, special attention was paid to tumors. Especially the appearances and morphology of melanomas, basal cell and squamous cell carcinomas in multiphoton imaging were described and criteria to discriminate those pathologies from healthy skin were derived [21,22,23,24,25]. Additionally, a commercial multiphoton tomograph for clinical applications became available indicating the success of *in vivo* dermal SHG and TPEF tomography. However, even though the specificity of NMSC detection is found to be excellent, the *ex vivo* discrimination between different cancer types needs some refinement [24]. In principle, it can be expected that the diagnostic accuracy can be improved upon inclusion of more spectroscopic information. Such complementary information can be provided by the addition of another optical contrast mechanism complementing the routinely applied SHG and TPEF. In this regard, reflectance-mode confocal laser scanning microscopy (RCLSM) [26,27,28] and fluorescence life-time imaging (FLIM) [21] were applied to aid skin cancer identification. While the implementation of RCLSM is inexpensive compared to multiphoton imaging, it provides morphological information which is principally already available from TPEF. In particular RCLSM provides no chemical information. FLIM, on the other hand, greatly supports spectrally resolved TPEF to distinguish fluorophores and provides information about the molecular environment of the fluorophores [29]. Unfortunately, the current implementations of FLIM are too slow—with dwell times in the range of 0.1 ms per pixel [30]—to be simultaneously applied with SHG and TPEF—with typical dwell times of 1 µs per pixel. 

Coherent anti-Stokes Raman scattering (CARS) microscopy is as fast as SHG and TPEF imaging but provides chemical contrast complementing the two other modalities: In CARS, molecular vibrations are the basis for the signal generation. Hence, CARS provides means to study the tissue morphochemistry. In a first step a pump and a Stokes photon, whose energy difference matches the energy of a molecular vibration, coherently drive selected molecular vibrations. In a second step, a probe photon is scattered inelastically off the driven molecular oscillators. In the last few years the combination of SHG, TPEF and CARS, *i.e.*, the application of multimodal imaging, proved to be a powerful tool for the diagnosis of various *ex vivo* cancers—e.g., brain cancer [31], head and neck squamous cell carcinoma [32] and breast cancer [33]. Moreover, first *in vivo* investigations on human skin using multimodal imaging were performed [34] and valuable reports about NMSC studies can be expected in the near future. To guide these *in vivo* studies the manuscript at hand focuses on the *ex vivo* appearance of NMSC—more precisely of basal cell carcinoma (BCC) and squamous cell carcinoma (SCC), which together account for 97% of all NMSC [1]. In this context the synergetic effect of multimodal imaging is highlighted and special attention is paid to CARS microscopy. To the best of our knowledge, by now only a single other report of *ex vivo* BCC surgery in multimodal imaging exists [35]. However, this study relies on a very limited number of samples and is restrained to only very small image areas. Furthermore, the described samples originated from Mohs surgery, which requires a different interpretation of the data.

## 2. Experimental

### 2.1. Sample Preparation

Skin samples of 14 patients (skin type I-III) have been collected in the course of a routine biopsy for diagnostic purposes (cancer samples, e.g., from the head, chest or abdomen) or originate from plastic surgery (healthy skin samples) at the Charité University Hospital Berlin (protocol approved by the Ethics Committee of the Charité University Hospital Berlin). Immediately after excision of the suspicious skin alteration the tissue is wrapped in a compress soaked with physiological NaCl solution. Without further delay the material is frozen onto a glass sample holder using cryo spray (08-Spray, Bio-Optica) and dipped into liquid nitrogen. Subsequently the samples are attached to a base of frozen distilled water and cut vertically to the skin surface into 20 µm thick sections using a cryomicrotome (Microm HM 560, MicromInternational, Walldorf, Germany). No embedding medium other than water is used and the temperatures of the base and blade are chosen to be −23 °C and −21 °C, respectively. The sections are deposited on CaF_2_ object slides (Crystal GmbH, Berlin, Germany) to avoid the non-resonant background occurring in CARS [36]. Without further treatment and cooling the samples are imaged. Parallel sections are prepared for the histopathological evaluation.

### 2.2. Experimental Setup

**Figure 1:**
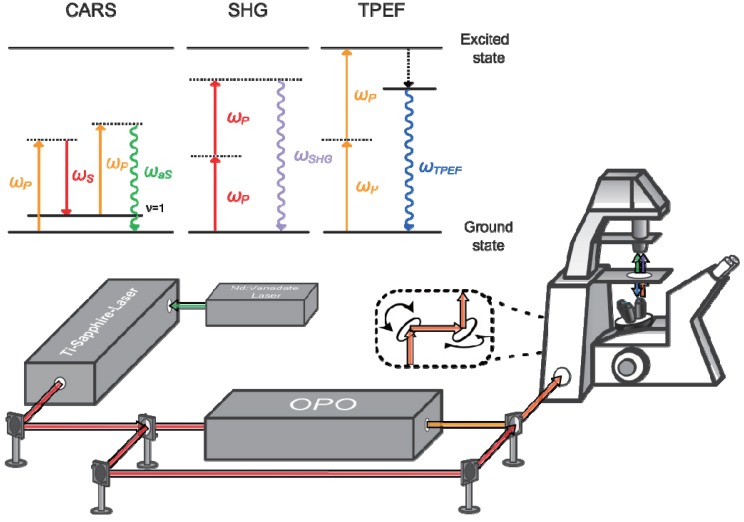
Experimental setup for the multimodal investigation of non-melanoma skin cancer (NMSC) and schematic representation of the three non-linear contrast mechanisms, *i.e.*, coherent anti-Stokes Raman scattering (CARS), two photon excited fluorescence (TPEF) and second harmonic generation (SHG).

The experimental setup used for non-linear multimodal imaging depicted in Figure 1 has been described previously [37]. Briefly, a continuous wave Neodymium-Vanadate laser with a power of 18 W operating at 532 nm is used to pump a Coherent Mira HP Titanium-Sapphire laser (Coherent, Santa Clara, CA, USA). The Titanium-Sapphire laser generates 2–3 ps pulses (FWHM) at a repetition rate of 76 MHz. The output of the laser at 830 nm is split into two parts. The first part is used directly, *i.e.*, without frequency conversion, as the Stokes beam, the second part is coupled into an optical parametric oscillator (OPO, APE, Berlin, Germany). The OPO provides wavelengths continuously variable in the range from 500 to 1,600 nm and is used as the pump beam. This allows for tuning the frequency difference of the pump and Stokes pulses at 671 nm to match the C-H symmetrical stretching vibration for the CARS measurements. Both beams are spatially combined by a dichroic filter and temporally overlapped using a mechanic delay stage equipped with a retroreflector. The joined laser beams are coupled into an inverse laser scanning microscope (LSM 510 Meta, Zeiss, Jena, Germany) and focused at the sample with a 20×/NA 0.8 achromatic objective (Zeiss).The signal detection is performed using photomultipliers (Hamamatsu Photonics, Hamamatsu, Japan) in forward direction (CARS, SHG) and backward direction (TPEF).Both single pictures and large area scans were recorded: The large area scans are composed of up to 15 × 15 tiles, each having a size of 450 µm × 450 µm with a resolution of 1,024 × 1,024 pixels. The pixel time is set to 1.6 µs. By averaging four scans the acquisition time for one tile does not exceed 16 s. Typically every sample is measured with two configurations, *i.e.*, the combination of CARS and TPEF signals as well as the combination of SHG and TPEF signals using only one laser beam. The repetition of measurement with a changed configuration was performed to optimize the excitation parameters for CARS and SHG separately, but is no necessity. To guarantee perfect co-registration of the images all three distinct signals, *i.e.*, CARS, TPEF and SHG could be detected simultaneous in future measurements. To access collagen’s fine structures in the proximity of tumor islands (see below) the detector gain for the SHG measurement is increased. All TPEF images depicted were observed using the first configuration optimized for CARS. Table 1 summarizes the experimentally relevant parameters.

**Table 1:** Overview of the experimental parameters.

Configuration	Excitation source	Central wavelength | FWHM/nm	Average power at the sample/mW	Peak irradiance at the sample/(W/cm^2^)
1st				
CARS @ 2,850 cm^−1^	OPO (Pump)	~671 | 0.6	~20	≈9 × 10^9^
TPEF @ 435–485 nm	Ti: sapphire	~830 | 0.5	~20	≈6 × 10^9^
	(Stokes)			
2nd				
SHG @ 415 nm	Ti: sapphire	~830 | 0.5	~80	≈2 × 10^10^

A discussion concerning average power, peak irradiance and photodamage thresholds can be found in reference [38].

A continuous wave laser source pumps the Ti-sapphire laser. The pulsed laser is split into two beams. Changing the frequency the first beam is coupled into an OPO and overlaid with the second beam. The joint beams are coupled into an LSM and scanned over the sample generating CARS, TPEF and SHG. In CARS the beating frequency of the pump (ω_P_) and Stokes (ω_S_) photon drives molecular oscillators. A probe photon (ω_P_) is inelastically scattered off the molecular oscillation and blue shifted. TPEF signal will be observed after simultaneous absorption of two photons (ω_P_) by a fluorophore, followed by relaxation processes and fluorescence emission. In contrast SHG is generated via simultaneous interaction of two photons with structures lacking inversion symmetry. 

### 2.3. Statistical Analysis

Statistical analysis of the images was performed with R [39] using the package png [40]. For every sample (5 BCC, 5 SCC) and each modality (CARS, SHG and TPEF) five pairs of 100 × 100 pixels sized areas have been selected manually for analysis. Each pair consists of a tumor area and a neighboring non-cancerous area for comparison. The selection of the areas was confirmed by a trained pathologist. Brightness differences between the tumorous and non-tumorous image have been investigated using the Wilcoxon matched pairs signed-rank Wilcoxon test, which tests whether two sets of observations come from the same population (null hypothesis H0) or differ in location (alternative hypothesis H1). The significance level was chosen to be 1%.

## 3. Results

The appearance of NMSC in multimodal imaging is investigated. Within this study more than 70 *ex vivo* sections of 14 patients are examined taking large overview scans of a maximum dimension of 6.75 × 6.75 mm and inter pixel distance of 440 nm, respectively. The 14 distinct samples of 14 patients comprise 5 nodular BCC, 5 well-differentiated SCC and 4 sections of healthy skin as confirmed by histological control. CARS images are recorded at a Raman shift of 2,850 cm^−1^ corresponding to the CH_2_ stretching vibration. At this vibration mainly CH_2_-rich lipids like fat stored in adipocytes [41], are imaged with minor contributions of proteins, which are more efficiently visualized at 2,930 cm^−1^ [42,43]. The SHG signal—collected best at wavelengths slightly above 800 nm [24,44]—maps the distribution of structures lacking inversion symmetry. In biological samples mainly structural proteins like collagen, myosin and tubulin fulfill this requirement [45,46,47]. However, due to its high abundance in skin and large first order non-linear susceptibility [48] collagen dominates the SHG images [49]. TPEF visualizes the distribution of native fluorophores. The skin autofluorophores detected at 435–485 nm, which contribute at the used excitation wavelength of 670 nm and 830 nm are melanin, NAD(P)H, flavins, keratin and elastin [44,50].

The joint application of CARS, TPEF and SHG—mapping molecular structures, fluorophores and supramolecular structures, respectively—provides compellingly rich detail on the skin’s morphochemistry. As the appearance of BCC in SHG and TPEF was described recently [22,24,30,51], this work focuses on CARS and highlights the interplay of the different modalities in multimodal imaging. The remainder of this paper is divided into three parts. (1) The appearance of BCC in the different modalities is characterized exemplarily on a section showing a nodular BCC; (2) An analogous representative analysis is demonstrated for a section of one SCC; (3) A statistical analysis of selected observations presented in Parts (1) and (2) is provided. Finally, the origin of these observations is discussed in detail.

### 3.1. Basal Cell Carcinoma

**Figure 2:**
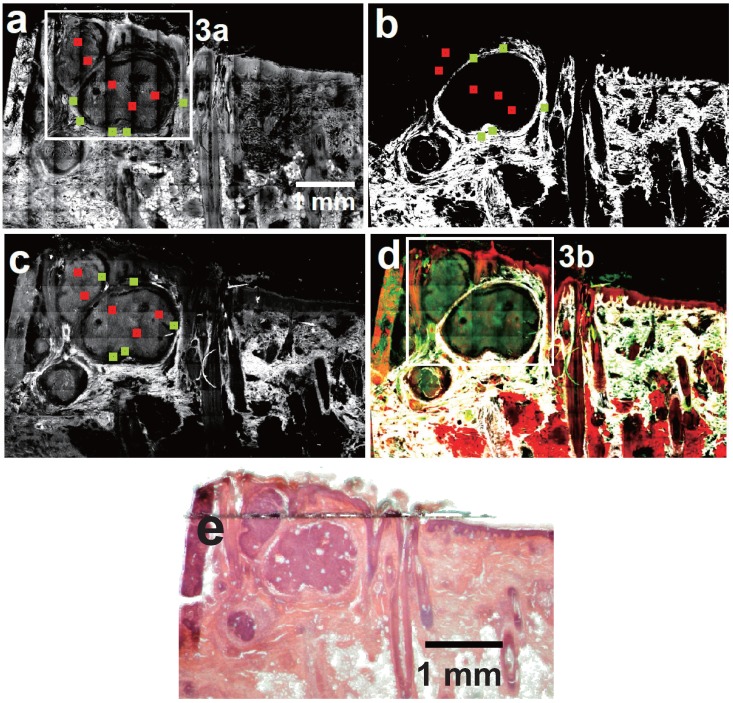
(**a**) CARS; (**b**) SHG; (**c**) TPEF (gray scale) and (**d**) multimodal image (CARS: red; TPEF: green; SHG: grays) as well as (**e**) H&E stained picture of the BBC section. The red (tumorous) and green (non-tumorous) squares locate the position of the 100 × 100 pixel sized areas selected for statistical analysis. (**a**) CARS overview image of a basal cell carcinoma (BCC). The BCC tissue appears darker in CARS. See also Figure 3a for image details encapsulated by the white box; (**b**) SHG image of the same BCC section. In general BCC tissue shows no SHG signal; (**c**) Corresponding TPEF image. BCC tissue appears slightly brighter than the adjacent dermal tissue, but darker than elastin fibers; (**d**) Multimodal large overview image, *i.e.*, the combination of the images 2a–c. See also Figure 3b for image details encapsulated by the white box; (**e**) H&E stained section.

**Figure 3:**
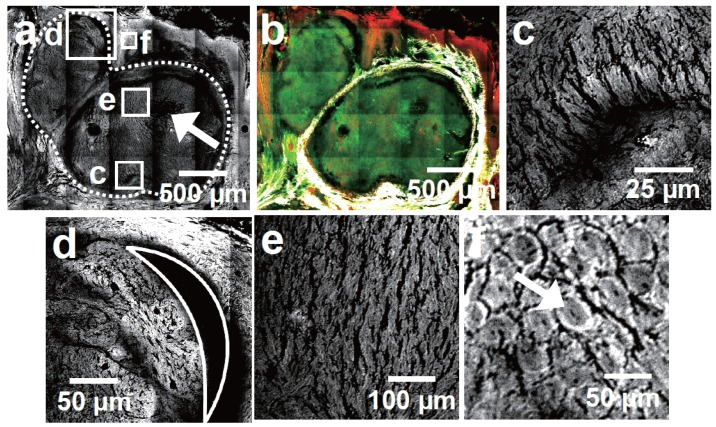
CARS and multimodal image details (CARS: red; TPEF: green; SHG: grays) taken from Figure 2a,d. (**a**) CARS image detail of the BCC tumorous region. The dotted line delineates the tumor nests. The arrow indicates the location of necrotic tissue; (**b**) Multimodal image of the tumorous region. The borders of the BCC tissue are unveiled by the SHG signal. A weak CARS and TPEF signal edging surrounds the tumor facilitating its localization; (**c**) Palisade shaped tumor border—an exclusive feature of BCC. The palisade shaped cells appear slightly brighter than the rest of the tumor nest in CARS; (**d**) Retraction artifact—typically shaped like a half-moon; (**e**) Tumorous tissue; (**f**) Non-tumorous epidermal tissue. Single cells as well as cell nuclei can be identified.

Figure 2 comprises a collocation of (a) CARS; (b) SHG; (c) TPEF and (d) the resultant multimodal images of a nodular BCC. For comparison Figure 2e displays an image of an H&E stained parallel section highlighting the histopathologically assigned tumor. Clearly, the tumorous tissue can be identified in the non-linear optical images (Figure 2a–d). Further, the three mayor layers of the skin, *i.e.*, the epidermis, dermis and subcutis as well as skin appendages, e.g., hair follicles, are distinguishable in each modality and best in the multimodal image as was highlighted in previous work [38]. The Figure 3a–f show image details of the Figure 2a,d.

Considering the data in detail, in Figure 2b it is found that the vicinity of the BCC tumor nests often depict a lower SHG signal than unaffected tissue. Furthermore, all larger tumor cell agglomerates (>100 µm) possess regions lacking of any SHG signal—see discussion. Instead, the tumor nests are enclosed by SHG signal and therefore the tumor borders are revealed. On the other hand, the tumor is characterized by a TPEF intensity that is lower than the TPEF signal of elastin fibers and some skin appendages, e.g., sebaceous glands, but higher than for the rest of the adjacent dermal tissue (see Figure 2c). Occasionally, a darker edge surrounding the relatively bright appearing tumor nests is observed in TPEF. Both SHG and TPEF observations are in agreement with recent literature studies [22]. Additionally, we can report here for the first time that BCC nests display a weak CARS signal intensity compared to the surrounding tissue (Figure 2a).

Except for intensity differences between healthy and cancerous tissue, morphological features—known from H&E staining libraries [52,53]—can be identified. Figure 3a,b depict the BCC tumor nests at higher magnification. Occasionally, larger tumor cell agglomerates display necrotic tissue [52] due to an insufficient nutrition supply. The white arrow in Figure 3a points to such a necrotic area, which is characterized by a low CARS and TPEF signal. In contrast to this general tumor feature, Figure 3c visualizes the BCC specific peripheral palisading [52]. In CARS these palisade-shaped cells enclosing the tumor itself appear slightly brighter than the rest of the tumorous tissue. Figure 3d shows BCC typical retraction artifacts [53] shaped like a half moon. Finally, Figure 3e,f compare the cellular structure of tumorous tissue with those of the epidermis. Interestingly, no individual cells can be identified inside the BCC tissue. Instead a bark-like texture is observed, which does not correspond to any finding in H&E stained samples. Most likely this observation can be ascribed to the modified sample preparation and might be a BCC-specific drying artifact. Five out of five BCC samples displayed this texture feature while only one SCC revealed a similar artifact. This observation might be ascribed to a distinct dehydration resistance of SCC and BCC due to their different point of origin (see discussion). In contrast to the lack of individual cells being observed in CARS images of BCC tissue, single cells are readily distinguishable within the epidermis. Here, even subcellular structures, such as the approximately 3 µm large nuclei, can be identified.

### 3.2. Squamous Cell Carcinoma

**Figure 4:**
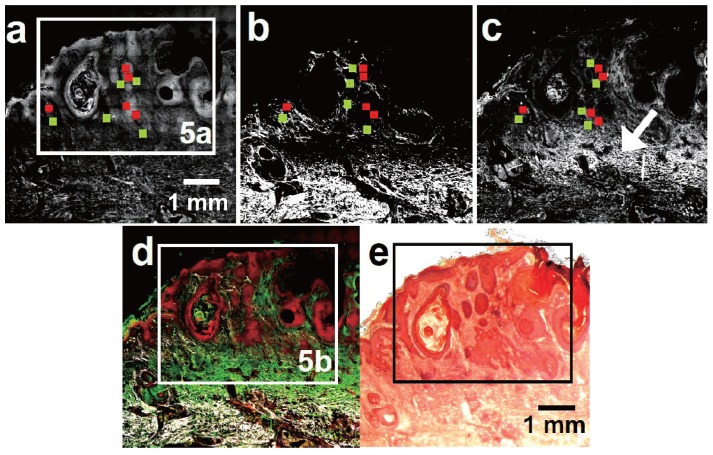
CARS, SHG, TPEF (gray scale) multimodal images (CARS: red; TPEF: green; SHG: grays) of a squamous cell carcinoma (SCC). The red (tumorous) and green (non-tumorous) squares locate the position of the 100 × 100 pixel sized areas selected for statistical analysis. (**a**) CARS overview image of the SCC; (**b**) Corresponding SHG image. Analogous to BCC, SCC tissue shows a negligible SHG signal; (**c**) TPEF image of the same region. Analogous to BCC tissue, SCC tumor nests show a slightly increased TPEF intensity compared to the adjacent dermal tissue. The white arrow points towards a layer of accumulated elastin fibers referred to as solar elastosis; (**d**) Multimodal image combining the information of Figure 4a–c; (**e**) H&E stained section of the SCC.

**Figure 5:**
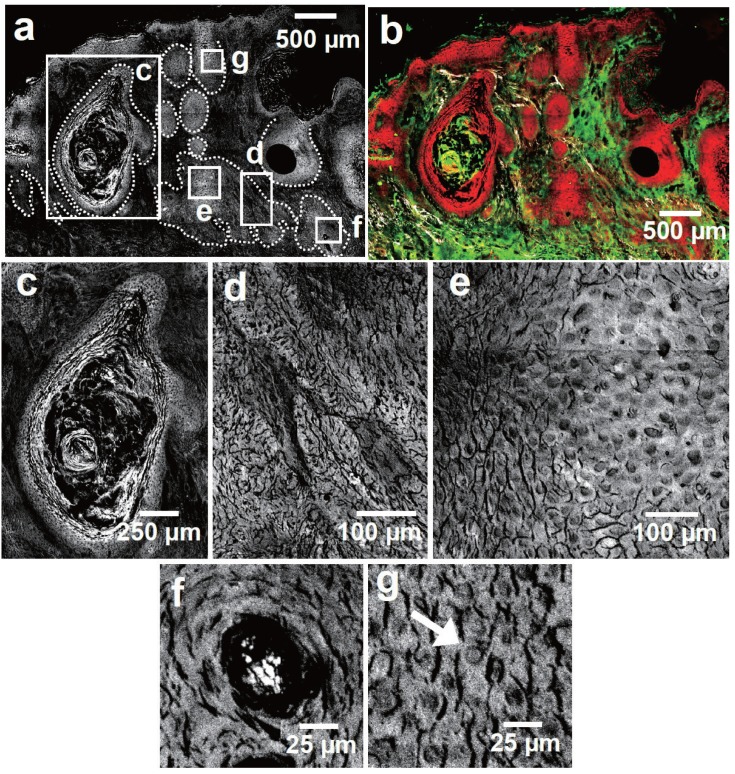
CARS (gray scale) and multimodal image (CARS: red, TPEF: green, SHG: grays) detail taken of Figure 4a,d. (**a**) and (**b**) CARS and multimodal image section illustrating the tumorous region, which is located by the dotted line; (**c**) Keratinizing tumor; (**d**) SCC tumor nest. The tumorous cells can be discerned from the embedding dermal tissue; (**e**) Tumor cells with pleomorphic nuclei; (**f**) Keratin pearl; (**g**) SCC cells. The white arrow points towards a nucleus appearing dark in CARS. Compared to non-cancerous tissue (see Figure 3f) the tumorous tissue possesses an increased cell density, larger nuclei and therefore an elevated nuclear cytoplasm ratio.

Having discussed the appearance of a BCC in multimodal imaging, Figure 4 depicts diagnostically relevant results of a SCC comprised as a collocation of (a) CARS; (b) TPEF; (c) SHG and (d) corresponding multimodal image. For comparison, Figure 4e shows the H&E stained parallel section illustrating the localization of tumorous SCC tissue. Again, the corresponding tumor cell agglomerates can be readily discerned. Further, morphological features—typical for SCC—are enlarged in the CARS and multimodal images shown in Figure 5.

As can be seen in Figure 4a,b, the SCC tissue shows up completely dark in the SHG image, while the tissue outside the tumor borders shows significant SHG intensities. In contrast, the tumorous tissue displays a relatively strong TPEF signal. Tumor exterior dermal constituents like elastin fibers and some skin appendages appear brighter in TPEF, whereas the adjacencies of the tumor nests display a weaker TPEF intensity than the tumor cell agglomerates (see also Image S2b). Additionally, in Figure 4c the large area with elevated TPEF intensity located distal to the tumor nests account for an accumulation of elastin fibers, which is referred to as solar elastosis [20,53]. In CARS these fibers do not stand out in terms of brightness [38]. On the contrary, the SCC tissue appears—opposed to BCC tissue—brighter in CARS than surrounding dermal constituents.

Furthermore, morphological features—routinely included in clinical NMSC diagnosis of H&E stained samples—can be identified using CARS: Figure 5c shows a well differentiated squamous cell carcinoma with marked keratinization infiltrating into the reticular dermis. Appearing even brighter in CARS a keratin pearl is depicted in Figure 5f [53]. The keratin pearl is enclosed by a tumor nest. The latter is displayed separately in Figure 5d,e. Clearly, Figure 5d shows tumor nests, which are less regular than for the BCC without palisading as expected for SCC [54]. Additionally, Figure 5e,g present the tumor islands in higher detail. Therein, cells of the size of about 20 µm and approximately 12 µm large nuclei are distinguishable. Moreover, the nuclei of the tumor cells are crowded, pleomorphic and of increased size compared to unaffected epithelial tissue exemplified in Figure 3f. 

## 4. Discussion

The results concerning the diagnosis of NMSC can be subdivided into morphological and intensity information. Diagnostically relevant morphological key features—illustrated in Figure 3 and Figure 5—are well-known from histopathological H&E staining. CARS is capable of revealing salient BCC and SCC features. Less intuitive, however, is the connection of the signal strength in multimodal imaging to staining techniques and, more importantly, its biochemical explanation. Therefore, the changed relative intensities for each modality should be analyzed in the following manner. 

All SCC and BCC tumorous cell agglomerates showed areas without SHG signal (see Figure 2b and Figure 4b as well as Table 2). Instead, the tumor islands are surrounded by SHG signal, which can be used to localize the tumor. This observation is in line with previous reports for BCC tissue [22] and in agreement with reports using collagen staining with, e.g., Sirius red [55]. Since also the malignant melanoma was described to display a very low SHG signal [56], this feature might be generalizable to the majority of skin cancers. However, a comprehensive explanation rationalizing this proposition is missing. Possibly, the origin of the tissue alteration can provide a starting point to rationalize these findings: BCC arise from malignant transformed cells of the basal layer of hair follicle or from sebaceous glands, while SCC cells are supposed to arise from keratinocytes of the *stratum spinosum* [57]. Neither the original basal cells nor keratinocytes produce collagen. Thus, it is rather unlikely that these cells gain the ability to produce large amounts of collagen during their malignant transformation, since the biosynthesis is a complex process involving intracellular, *i.e.*, the production of tropocollagen, as well as extracellular transformation steps, which are usually only achieved by fibroblasts [58]. 

**Table 2:** *p*-values of the one-sided paired Wilcoxon signed-rank test with H_0_ given in parenthesis. The location shift µ denotes the difference in total intensities of the corresponding neighbor and tumor areas I_neighbor_ and I_tumor_, respectively, *i.e.*, µ = I_neighbor_ − I_tumor_.

*p*-values (H_0_)	CARS	SHG	TPEF
BCC	2.086 × 10^−7^ (H0: µ < 0)	2.98 × 10^−8^ (H0: µ <0)	2.98 × 10^−8^ (H0: µ > 0)
SCC	9.711 × 10^−6^ (H0: µ > 0)	2.98 × 10^−8^ (H0: µ <0)	2.98 × 10^−8^ (H0: µ > 0)

**Figure A1:**
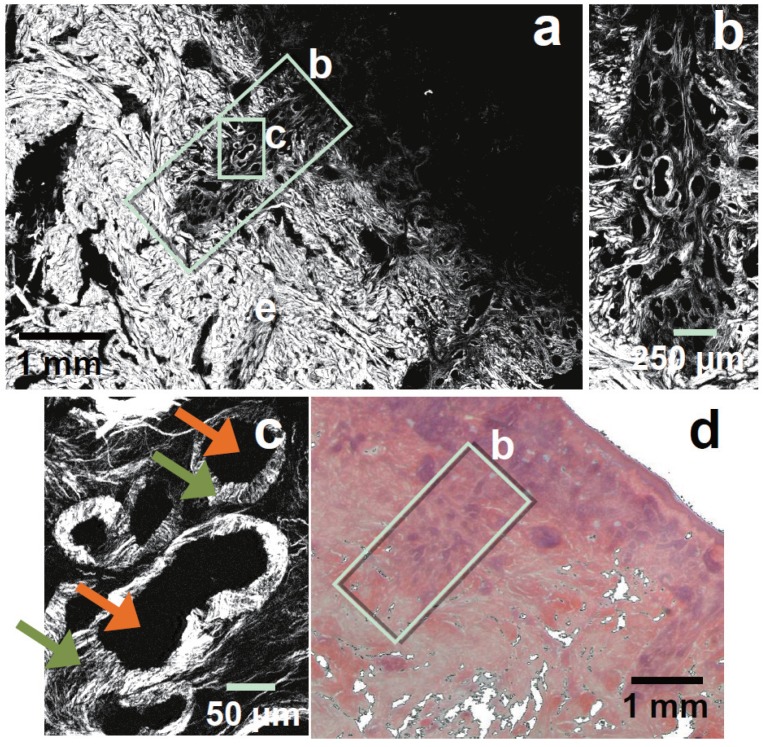
SHG images of an invasively growing BCC with peculiar collagen fiber orientation. (**a**) Overview scan of the BCC; (**b**) Infiltrate growing area; (**c**) Tumor nests. The orange arrows point at the BCC tumor nests lacking any SHG. The green arrows point to positions with collagen fibers, which are directed dominantly perpendicular to the tumor nest surface; (**d**) H&E stained parallel section with marked tumorous region.

In addition, the interaction of skin cancers with the extracellular matrix has been the subject of numerous investigations [59], since the tumor migration—a crucial step in the tumor invasion-metastasis cascade [60]—is deeply connected to the equilibrium between proteolysis and reconstruction of the connective tissue [57,61]. Thereby, the decreased SHG intensity in the proximity up to several 100 µm outside the NMSC tumor nests can be explained (see Figure 4b and Figure A1b). In turn the evaluation of the SHG contribution in multimodal images might deliver valuable prognostic information. To give an example—one invasively growing BCC possessed an unusual perpendicular orientation of collagen fibers towards the tumor surface (see supplementary information and Figure A1a–c), which was also reported for invasive mammary tumors [62]. To evaluate the prognostic benefit of these tumor-associated collagen signatures (TACS) further investigations are necessary. A similar discussion for the collagen distribution visualized by SHG could be provided for the elastin appearing prominently in TPEF. For conciseness reasons this shall be omitted and can be found in the supporting information.

Furthermore, the locally increased TPEF intensity inside BCC and SCC tumors with dark edges surrounding the tumor nests will be considered in some detail. Previous investigations with comparable experimental conditions reported, *i.e*., TPEF signal detection in the range of 435–485 nm, NADH and NADPH to be the autofluorophores (NAD^+^ and NADP^+^ are non-fluorescent [63]) with the strongest fluorescence signal within living skin cells [64]. Both coenzymes are intrinsic biomarker for mitochondrial anomalies as well as metabolic activities [63]. Especially an altered concentration of NADH compared to non-cancerous tissue was reported for different tumors, e.g., breast cancer [65,66] and malignant tissue in the oral cavity [65]. The local concentration of NADH is supposed to be a measure of energy respiration [50], which is frequently increased in cancer cells due to their accelerated metabolism. The increased TPEF intensity within BCC and SCC can hence be rationalized. Thus, the TPEF activity of malignant cells might be an interesting parameter to estimate the growth speed in order to provide a reasonable prognosis and an appropriate treatment. For an accurate evaluation of the NADH content it has to be considered that the cellular NADH level is reported to decrease up to 4 h after tissue resection [67]. Translating this observation into an expectation for *in vivo* examinations the NADH decrease over time might result in even higher TPEF contrast between *in vivo* malignant and non-malignant tissue. 

In contrast to NADH the lipid content and constitution of the excised section—being imaged by CARS—is assumed to reflect the *in vivo* scenario. Interestingly, BCC and SCC tumor nests differ in terms of their CARS intensity compared to the surrounding non-tumorous tissue (see Figure 2a, Figure 3a, Figure 4a and Figure 5a). While BCC cell agglomerates appear relatively dark in CARS, SCC tissue appears brighter in comparison to adjacent collagen containing tissue. The reason for this observation is unclear up to now and leaves room for speculations. In general, the distinct appearances of BCC and SCC have to be related to either the constitutional properties of the original cells, which are partially maintained (1), or otherwise the new appearance was gained under malignant transformation (2) or both. (1) is supported by the fact that SCC arises from higher differentiated epithelial cells than BCC [57] with a relatively increased amount of lipids [68] and proteins, such as keratin and filaggrin [69], which would cause the observed higher CARS signal. In contrast, it is known that cancer cells show a changed lipid metabolism [70]. The alteration of lipid anabolism and catabolism affects numerous processes including cell proliferation, growth, differentiation and motility [71] rendering the lipid content an increasingly recognized hallmark of cancer [72]. Even though understanding the influence of lipids towards cancer development is just at its beginning [73], attention towards dysregulated lipid metabolism increased over the last decade [71,72]. It is reported that tumor cells do not store significant amounts of triglycerides [74], which could explain the low CARS intensity for BCC tumor nests. On the other hand cancer cells show elevated levels of free fatty acids (FFA) [72], which has been found to be associated with a poor prognosis [75]. Excess of FFA incorporated into the cell membrane of cancer cells are supposed to mediate tumor metastasis by reducing cell-cell contact and promoting tissue invasion [76]. Therefore, an increase of the FFA level would justify the relatively stronger CARS intensities of the investigated SCC and, moreover, give reason for the generally higher affinity of SCC to metastasize compared to BCC [77]. Final conclusions in this respect, however, are not the scope of this work. 

In addition to the visual detection and individual interpretation of NMSC, a major goal of multimodal imaging is to provide a computerized tool that facilitates NMSC diagnosis. An intermediate step towards computerized NMSC detection is to prove whether the intensity differences used for the visual discrimination between tumorous and non-tumorous tissue are statistically significant. Thus, to evaluate the observed intensity differences between the tumorous and non-tumorous tissue a one-sided paired Wilcoxon signed-rank test is applied to all NMSC. For each modality five paired (tumorous and non-tumorous) sections of 100 × 100 pixels are chosen. The tumorous areas are selected arbitrarily from the histopathologically assigned tumor nest regions. For CARS and SHG the corresponding non-tumorous tissue originate from collagen containing tissue next to the selected tumorous areas. For TPEF, however, the non-tumorous tissue sections arise from dermal tissue next to tumor nests, which contains no elastin. To illustrate the choice of sections the small red (tumorous) and green (non-tumorous) squares in the Figure 2a–c and Figure 3a–c exemplify the selection. The result of the Wilcoxon test is presented in Table 2. 

The Wilcoxon test shows that the selected tumorous and non-tumorous areas are clearly distinguishable via their significantly different intensities. However, due to the polymorphism of the skin with its numerous layer and appendages, as well as the size of each image the computerization of NMSC diagnoses in multimodal images, remains a challenge. Future research concerning the automated NMSC detection might use the derived visual distinguishing marks and focus on the inter-patient variance investigating larger sample sets. To accomplish this, an experimentally stable and alignment free setup [78] for minimized inter-image variance as well as an increased imaging speed, is necessary to investigate a statistically sufficient number of samples in reasonable time. 

**Figure 6:**
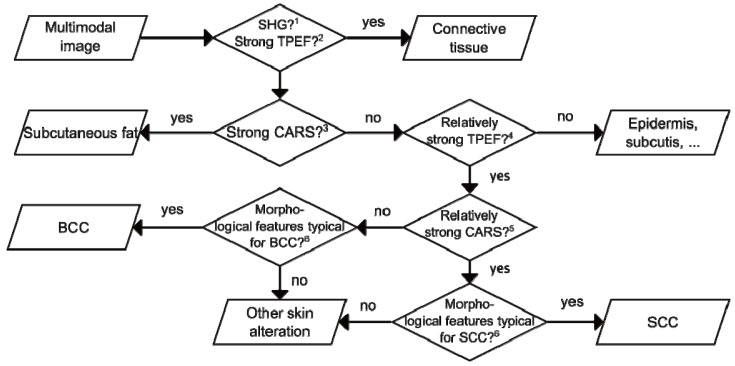
Flow chart suggesting a pathway for manual NMSC diagnosis analyzing multimodal images. It might form the basis for a future computerized discrimination of NMSC and healthy tissue. For a detailed explanation see the note.

Note: ^1^ NMSC displays no SHG signal. Collagen is responsible for most of the SHG signal in skin; ^2^ The strongest TPEF signal is caused by elastin, which is not observed in NMSC; ^3^ The strongest CARS signal is emitted by fat due to the high concentration of CH_2_-groups; ^4^ NMSC tissue displays a relatively strong TPEF signal compared to its adjacencies except for elastin; ^5^ It was found that BCC and SCC tissue differ in terms of their CARS signal strength. The BCC tissue appears relatively dark compared to the surrounding collagen containing tissue, while the SCC tissue appears comparably bright; ^6^ Features that are well known from routinely applied histopathological staining.

Having discussed the origin of the observed NMSC morphochemistry profiles, a simple guideline for the interpretation of multimodal images towards tumor diagnosis might be derived. This approach is schematically summarized in Figure 6. First, a suitable region of interest is selected, *i.e.*, the 6.3 × 4.1 mm^2^ and 6.3 × 6.3 mm^2^ large images shown in Figure 2b and Figure 3b, respectively. Areas with a SHG or a strong fibrously shaped TPEF signal indicate the presence of collagen or elastin, respectively. As a result, these regions can be ignored for tumor recognition. Analogously, regions with a dominating CARS signals point to subcutaneous fat and can therefore be assigned to non-NMSC tissue. Next, the relative TPEF signal intensity is analyzed. The investigated tumorous tissue shows a clear tendency to depict a slightly stronger TPEF intensity than the adjacent dermal tissue, but a lower TPEF activity than elastin fibers and some skin appendages like hair follicles and sebaceous glands [38]. To discriminate BCC and SCC the relative CARS intensities are considered in the next step. While BCC tissue appears dark, SCC tissue shows a relatively higher CARS signal than collagen containing non-tumorous tissue. Finally, the classification of the identified suspicious regions has to be confirmed by taking morphological features into account (Figure 3 and Figure 5), which are predominantly—or exclusively—found in either SCC or BCC. 

The multimodal NMSC analysis procedure reported here, which combines relative intensity considerations and morphological information, was evaluated manually by investigating three samples of unknown malignancy. All three samples were correctly classified as BCC, SCC, or healthy tissue. 

## 5. Conclusions

The *ex vivo* appearance of non-melanoma skin cancer (NMSC) was analyzed using an all optical multimodal approach, *i.e.*, the joint application of coherent anti-Stokes Raman scattering (CARS), second harmonic generation (SHG) and two photon excited fluorescence (TPEF). Both basal cell (BCC) and squamous cell carcinoma (SCC) show a lack of SHG and increased TPEF signal for cancerous tissue. Moreover, the distinct appearance of BCC and SCC in CARS is reported. While the BCC regions display a relatively weak CARS signal, the SCC nests show a strong CARS signal compared to the surrounding tissue. Additional to the delineation of the tumor nests, prognostically relevant information about the lipid metabolism, bioenergetics and tumor-stroma interaction is at hand by evaluation of the multimodal images. Furthermore, cancer-type characteristic morphological features using H&E staining can be identified. These are an increased cell density, enlarged nuclei, a changed nuclear cytoplasm ratio for SCC as well as a palisade shaped tumor border, retraction artifacts and central necrosis for BCC. The combined information clearly allows to differentiating healthy skin from NMSC. Moreover, it provides evidence to distinguish BCC and SCC, as well as a means to evaluate parts of the tumor biochemistry.

## Figures and Tables

**Figure A2:**
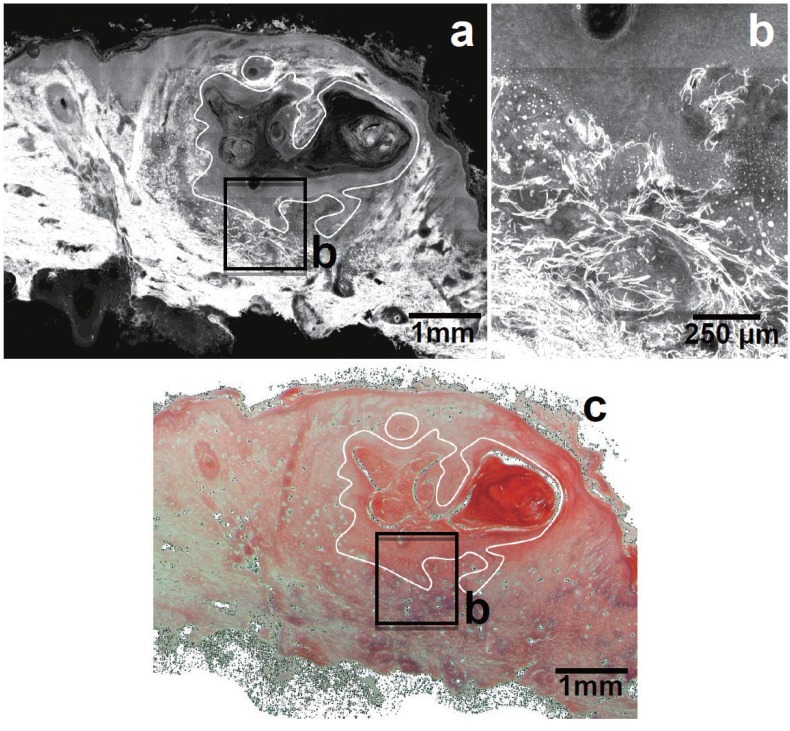
TPEF images of a SCC. (**a**) Overview scan of a SCC. The white curve illustrates the tumor border; (**b**) Tumorous tissue adjacent to non-tumorous tissue. The elastin fibers appearing brightest in TPEF are located exclusively in the non-tumorous area; (**c**) H&E stained section with marked tumorous region.

## Appendix

### Tumor-Associated Collagen Signatures

A single invasively growing BCC in this study (in total 5 BCCs were investigated) showed lots of collagen fibers, which are oriented perpendicular towards the tumor nest surface (Figure A1). In contrast, non-invasively growing BCC show a collagen fiber orientation predominantly parallel to the tumor nest surface.

### Elastin Fiber Distribution in NMSC

In analogy to the collagen distribution, larger tumor nests (>100 µm) show regions without any elastin fibers (see Figure A2), which belong to the strongest TPEF sources of the skin and are therefore readily distinguishable. Often—but less reliably than for collagen in SHG—the presence of elastin fibers outlines the borders of the tumorous tissue.
